# Modeling the Keys to Team’s Success in the Women’s Chinese Basketball Association

**DOI:** 10.3389/fpsyg.2021.671860

**Published:** 2021-06-04

**Authors:** Qing Yi, Shaoliang Zhang, Wenxuan Fang, Miguel-Ángel Gómez-Ruano

**Affiliations:** ^1^School of Physical Education and Sport Training, Shanghai University of Sport, Shanghai, China; ^2^Shanghai Key Lab of Human Performance, Shanghai University of Sport, Shanghai, China; ^3^Division of Sport Science & Physical Education, Tsinghua University, Beijing, China; ^4^Facultad de Ciencias de la Actividad Física y del Deporte (INEF), Universidad Politécnica de Madrid, Madrid, Spain

**Keywords:** women, team sports, performance analysis, match analysis, quantile regression

## Abstract

The technical characteristics of women’s basketball may differ from men’s basketball, and there is a need to identify the key performance indicators (KPIs) that contribute to the success of women’s teams. The aim of the current study was to examine and quantify the relationships between technical performance indicators and match outcome in elite women’s basketball using both linear and non-linear statistical methods, the effectiveness of the two methods was compared as well. A total of 136 matches (*n* = 272 teams’ observations) in the regular season of Women’s Chinese Basketball Association (WCBA; season 2020–2021) were analyzed using multiple linear regression (MLR) and quantile regression (QR). Results showed that two-point percentage, offensive rebounds, assists and turnovers had significant effects on the match outcome for both MLR and QR analysis. No significant relationships were observed between match outcome and three-point percentage, steals, and fouls. The results between MLR and QR analysis were different in free-throw percentage, defensive rebounds and blocks. Current results highlighted QR analysis is an advanced statistical model more powerful than the traditional linear method for the identification of KPIs. The identified KPIs may help coaches to develop more specific training interventions and match strategies during match play.

## Introduction

One of the most important tasks of performance analysis in basketball is to interpret and quantify the dynamical interactions among technical, tactical, physical and psychological factors during matches (Drust, 2010). Over the years, researchers have paid more attention on the technical match performance of men’s basketball, especially in the identification of key performance indicators (KPIs) (Gómez-Ruano et al., 2008; Lorenzo et al., 2010) and tactical patterns (Kempe et al., 2015), the effect of situational variables (Sampaio et al., 2010; García et al., 2014), and the use of performance profiling (Zhang et al., 2017, 2019). However, the available literature regarding the exploration of women’s technical match performance is scarce (Gómez-Ruano et al., 2006, 2013; Leicht et al., 2017a). The sex differences should not be ignored when choosing the research object, as differences in technical (Sampaio et al., 2004), anthropometric (Garcia-Gil et al., 2018), and physiological (Scanlan et al., 2012) characteristics may exist between men and women’s players. Previous research has reported that women differ from men in the effectiveness of collective movement patterns during match play (Gómez-Ruano et al., 2013), and women’s teams obtained higher unsuccessful two-point field-goals and steals, and lower blocks than men’s teams (Sampaio et al., 2004).

The technical match performance could be interpreted and quantified by a set of technical performance indicators (Liu et al., 2016), and the technical performance indicators is a combination of match actions and events that aims to explain some or all aspects of a successful match performance (Hughes and Bartlett, 2002). Therefore, in light of the gender differences in technical performance indicators (e.g., steal and block) and well-documented literature for men’s basketball, it would be of interest to identify the KPIs that best can explain the match characteristics of women’s basketball. Gómez-Ruano et al. (2006) employed the discriminant analysis to identify the KPIs that better differentiate winning and losing teams, where successful two-point field-goals, defensive rebounds and assists were identified as the key predictors for women’s basketball matches. Another study from Leicht et al. (2017a) employed both linear and non-linear statistical models to examine the relationships between technical performance indicators and the match outcome for women’s basketball matches at the Olympic Games. Field-goal percentage, defensive rebounds, steals, and turnovers were considered as the key indicators of match outcome, concluding that the combination of distinctive KPIs with the non-linear modeling could provide teams with a greater likelihood of winning a match. It is therefore suggested that the non-linear statistical techniques could be useful tools for coaches and performance analysts in the evaluation of teams’ and players’ match performances.

The current study proposed a novel non-linear statistical model called quantile regression (QR), developed by Koenker and Bassett (Koenker and Bassett, 1978; Koenker, 1994), to identify the relationships between technical performance indicators and match outcome, these relationships were considered as linear and estimated by linear equations in the previous studies. However, the technical data collected from basketball matches cannot meet the conditions of traditional linear regression (e.g., linearity, homoscedasticity, independence, or normality) in most cases, especially for analyses with a limited sample size (Montgomery et al., 2021), whereas QR modeling provides that capability. Besides, traditional linear regression summarizes the average relationship between a set of independent variables and dependent variable based on the conditional mean function (Bilgili et al., 2021), but fails to fully capture the patterns in the data and may only provide a partial view of the relationships. Conversely, the QR modeling performs a stratified analysis and describes the relationships at different points in the conditional distribution of the dependent variable, enabling examination of the relationships between various conditional quantiles (e.g., 10th, 25th, 50th, 75th, and 90th quantiles) of the dependent variable and independent variables (Koenker et al., 2017). Therefore, the heterogeneities among the relationships could be revealed with QR modeling. Zhang et al. (2020) compared the effectiveness between multiple linear regression (MLR) and quantile regression for identifying the KPIs of men’s basketball matches at the FIBA Basketball World Cup, reporting that QR modeling explored additional KPIs (mid-range score at 10th quantile and offensive rebounds at 90th quantile) than MLR modeling. Therefore, QR modeling could be considered as a potentially superior tool for performance analysts to explain the match performance based on multivariate datasets.

The aim of this study was to identify the relationships between match-related statistics and match outcome in elite Women’s basketball teams (Women’s Chinese Basketball Association, WCBA), using linear and non-linear modeling. We hypothesized that QR modeling would allow us to identify the KPIs that can better explain the technical characteristics within matches and provide more detailed information for quantifying the relationships between KPIs and match outcome in elite women’s basketball.

## Materials and Methods

### Data Source and Reliability

Technical match performance data of teams from the regular season of the WCBA in the season 2020–2021 were acquired from the official website of the Chinese Basketball Association^1^. The reliability and accuracy of data were tested by two experienced analysts with more than 5 years of experience in basketball performance analysis using a randomly selected sub-sample of 10 matches. The test results were compared with the corresponding data from the official website and acceptable Intra-Class Correlation Coefficients (ICC = 0.87–0.98) were obtained for all variables. This study used a observational design and all the analyzed data were de-identified and available in the public domain, no stipulations were in place from the WCBA regarding re-use of the data for production of scientific manuscripts without permission, so ethics approval was not required, but the study design and procedures were in accordance with the ethical guidelines of the authors’ affiliated institutions.

### Sample and Technical Variables

There were 17 teams participating in the regular season of WCBA in season 2020–2021, with each team played against the other 16 teams one time. The teams’ technical match-related statistics of all 136 matches (*n* = 272 team observations) were selected as the sample. After disregarding the effect of multicollinearity among the explanatory variables, ten technical variables were analyzed and classified into two groups, offensive and defensive variables, according to previous studies (Gómez-Ruano et al., 2009; Sampaio et al., 2015; Zhang et al., 2020). The grouping information and operational definitions of these technical variables are presented in Table 1. The normalization of all variables was performed using the number of ball possessions (Ibáñez et al., 2009b; Gómez-Ruano et al., 2015). Ball possession was defined as a period of play between when one team gains the control of the ball and when another team gains the control of the ball (Sampaio and Janeira, 2003). The equation for calculating the ball possessions was as follows: ball possessions = field goals attempted – offensive rebounds + turnovers + 0.44 × free throws attempted (Oliver, 2004; Kubatko et al., 2007).

**TABLE 1:** Selected technical performance-related match variables.

Groups	Variables: operational definitions
Offensive variables	**Two-point percentage:** the percentage of two-point field goal attempts that were successful during the match
	**Three-point percentage:** the percentage of three-point field goal attempts that were successful during the match
	**Free throw percentage:** the percentage of free throws that were successful during the match
	**Offensive rebound:** the number of rebounds a player or team collected while on offense
	**Assist:** an assist occurs when a player completes a pass to a teammate that directly leads to a field goal score
	**Turnover:** a turnover occurs when the player or team on offense loses the ball to the defense
Defensive variables	**Defensive rebound:** the number of rebounds a player or team collected while on defense
	**Steal:** a steal occurs when a defensive player takes the ball away from a player on offense
	**Block:** a block occurs when the defense player tips the ball and prevents an offensive player’s shot from scoring
	**Foul:** any infringement that is penalized as foul play by a referee

### Statistical Analysis

Previously, the MLR has widely been used by researchers to identify the relationships between KPIs and the match performance of players and teams (Liu et al., 2016; Yi et al., 2019a,b). However, the traditional MLR method was modeled based on the average relationships between the dependent variable and a set of independent variables using the conditional mean function (Koenker and Bassett, 1978). This kind of mean regression modeling presumes that the dependent variable could be interpreted as a linear combination of a set of independent variables, but the level of the dependent variable has not been considered. It cannot estimate the overall impact of explanatory variables on the explained variables, only an average effect provided. QR describes the relationships between dependent and independent variables at different points of the conditional cumulative distribution of the dependent variable, and produces different coefficients for each prespecified quantile (decile or centile) of the error distribution (Koenker and Bassett, 1978). It enables researchers to understand the entire distribution of measured correlations conditional on a set of explanatory variables. Given that the sample contains non-normal disturbances, applying the conditional mean estimators to the main equation would not be suitable because these estimators are not robust to departures from normality or long tail error distributions. Hence, MLR is likely to produce inefficient and biased estimates. In contrast, the QR as a conditional median approach is robust to departures from normality and skewed tails (Bilgili et al., 2021).

In the current study, MLR and QR were both employed to identify the relationships between technical variables and match outcome, and the results between these approaches were compared. The examination of data distribution and multicollinearity were conducted before analyzing the effects of KPIs on the match outcome (final point differential) using MLR and QR models, respectively. The MLR and QR modeling were performed using R software (R project version 3.5.1). QR modeling was denoted as Qq(*y*/*x*), where *q* is the quantile or percentile, the median is the 50th percentile of the empirical distribution with no zero values for the dependent variable (Koenker, 1994). The relationships were interpreted by the positive and negative regression coefficients, which indicate a greater/lower propensity to increase/decrease the match outcome (Sampaio et al., 2010). The current study selected five quantile levels (Q10, Q25, Q50, Q75, and Q90) for the QR model. Q10 and Q25 represent the lower tail distribution and Q75 and Q90 represent the higher tail distribution. The statistical significance was set at *p* < 0.05.

## Results

**FIGURE 1:**
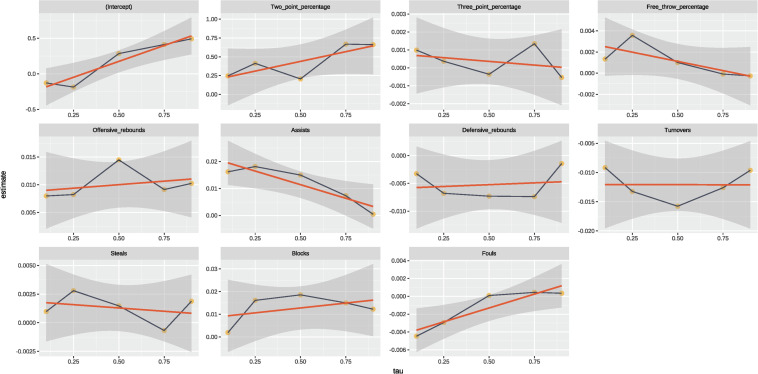
Regression coefficients of MLR and QR modeling for the effects of key performance indicators on match outcome.

**FIGURE 2:**
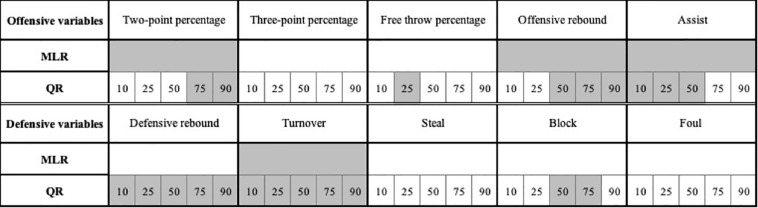
Comparison between MLR and QR modeling for the identified key performance indicators.

The parameter estimates of the MLR and QR with five quantile levels (Q10, Q25, Q50, Q75, and Q90) are shown in Table 2. Figure 1 is the visualization combining the results of MLR and QR modeling, and the significant technical indicators for both approaches are summarized and compared in Figure 2.

**TABLE 2:** Parameter estimates from multiple linear regression (MLR) and quantile regression (QR) on the difference quantiles of final score.

Variables	Multiple linear regression	Quantile regression (QR)				
		
		Q10	Q25	Q50	Q75	Q90
		(*n* = 272)	(*n* = 272)	(*n* = 272)	(*n* = 272)	(*n* = 272)
Constant	0.175 (0.152)	−0.129 (0.189)	−0.187 (0.205)	0.284 (0.179)	0.411* (0.201)	0.492** (0.132)
Two-point percentage	0.517** (0.173)	0.246 (0.200)	0.413 (0.229)	0.206 (0.202)	0.668** (0.216)	0.662** (0.193)
Three-point percentage	0.001 (0.002)	0.001 (0.002)	0.0004 (0.002)	−0.0004 (0.002)	0.001 (0.002)	−0.001 (0.001)
Free-throw percentage	0.001 (0.001)	0.001 (0.002)	0.004* (0.002)	0.001 (0.001)	−0.0001 (0.001)	−0.0003 (0.001)
Offensive rebound	0.011** (0.003)	0.008 (0.005)	0.008 (0.005)	0.014** (0.003)	0.009* (0.004)	0.010** (0.004)
Assist	0.011** (0.003)	0.016** (0.006)	0.018** (0.004)	0.015** (0.003)	0.007 (0.004)	0.0005 (0.004)
Defensive rebound	−0.006 (0.003)	−0.003 (0.003)	−0.007* (0.003)	−0.007* (0.003)	−0.007* (0.003)	−0.001 (0.003)
Turnover	−0.013** (0.003)	−0.009* (0.004)	−0.013** (0.003)	−0.016** (0.004)	−0.013** (0.004)	−0.010** (0.003)
Steal	0.0001 (0.004)	0.001 (0.006)	0.003 (0.005)	0.001 (0.004)	−0.001 (0.005)	0.002 (0.004)
Block	0.012 (0.007)	0.002 (0.014)	0.016 (0.010)	0.019** (0.007)	0.015* (0.007)	0.012 (0.010)
Foul	−0.002 (0.003)	−0.004 (0.004)	−0.003 (0.004)	0.0001 (0.004)	0.0004 (0.004)	0.0003 (0.003)

Standard errors are shown within parentheses; * *p* < 0.05 and ** *p* < 0.01.

The horizontal axis presents the different quantiles, the vertical axis lists the regression coefficients. The black line with yellow dots is the estimate of the regression coefficient for quantiles (10th, 25th, 50th, 75th, and 90th), the red line represents the corresponding regression coefficient of MLR. The light orange and deep gray shaded areas represent the 95% confidence intervals of the regression coefficients for QR and MLR, respectively.

### Offensive Variables

There was a significantly positive relationship between two-point percentage and the match outcome (regression coefficient, RC = 0.517) for MLR, while the significantly positive relationships were only found at the quantile of 75th and 90th (RC = 0.668 and 0.662) in the QR analysis. No significant relationship was detected between three-point percentage and match outcome for both MLR and QR modeling. Free-throw percentage also had no evident impact on the match outcome for both models, except for the 25th quantile (RC = 0.004). Offensive rebounds showed significantly positive relationships with match outcome for MLR (RC = 0.011) and QR with quantiles of 50th, 75th, and 90th (RC = 0.014, 0.009, and 0.010). A significantly positive relationship between assists and match result was identified for MLR (RC = 0.011) and QR with the quantiles of 10th, 25th, and 50th (RC = 0.016, 0.018, and 0.015).

### Defensive Variables

There was no significant relationship between defensive rebounds and match outcome for MLR, but the significantly negative relationships were found for QR analysis with the quantiles of 25th, 50th, and 75th (RC = −0.007, −0.007, and −0.007). Turnovers showed significantly negative relationships with the match result for MLR (RC = −0.013) and all QR quantiles (RC = −0.009, −0.013, −0.016, −0.013, and −0.010). No significant relationships between steals and match result were found for MLR or QR analyses with all quantiles. Blocks showed significantly positive relationships with match result at the quantiles of 50th and 75th for QR analysis (RC = 0.019 and 0.015), while no significant relationship could be detected for the MLR analysis. Fouls had no evident effect on the match result based on the results from both statistical approaches.

## Discussion

This study aimed to identify the relationships between technical performance indicators and match outcome in the WCBA, quantifying the effects of KPIs on the match outcome using MLR and QR models. Our results showed that offensive variables (two-point percentage, free-throw percentage, offensive rebound, and assist) had positive effects on the match outcome, while the defensive variables showed both positive and negative effects on the match outcome. The differences in the results between MLR and QR were identified, with QR analysis providing more detailed information for the quantification of the relationships between KPIs and match outcome.

Previous studies have reported that two-point percentage is the critical indicator for basketball match performance (Lorenzo Calvo et al., 2010; Parejo et al., 2013), and most of the points scored in a basketball game through two-point field goals (Ibáñez et al., 2009a). Our results indicated that two-point percentage had the greatest impact on the match result for MLR and QR analysis, a one-unit increase in two-point percentage would bring an increase of 0.517 units (MLR), 0.668 units (75th quantile) and 0.662 (90th quantile), respectively, for the match outcome. Scoring in the paint and mid-range area means that more offensive actions need to perform, and more physical contact with defenders would face (Gasperi et al., 2020; Reina et al., 2020). Therefore, more effective offensive actions that lead to two-point field goals, such as dribble penetration or post play, and greater physical ability would heighten the likelihood of team success. Unexpectedly, we found that three-point percentage had no significant relationship with the match outcome, which is inconsistent with a prior study from Zhang et al. (2020) who reported that three-point score was a KPI that significantly associated with match outcome for men’s basketball matches at the FIBA World Cup. This may be partly explained by the differences in the anthropometrical characteristics between men’s and women’s players (Garcia-Gil et al., 2018). The relative less strength and height may be a disadvantage for women’s players to reach higher accuracy in the three-point field goal given the longer distance that must be covered with shots compared to two-point field goals (Miller and Bartlett, 1996). The positive effect of strength on the accuracy of three-point field goals has been confirmed by previous studies (Tang and Shung, 2005; Justin et al., 2006). Therefore, frequency of occurrence of three-point field goal in women’s matches may be relatively lower than in men’s matches. Free-throw is executed under much more controlled and stable conditions than field goals, and the shooting accuracy is influenced by limited factors. It was considered as one of the most effective scoring methods, especially the importance at the last 5 min in close matches has been previous highlighted (Kozar et al., 1994). A prior study of women’s basketball from Gómez-Ruano et al. (2006) identified that free-throw percentage was not a KPI associated with match outcome for all matches and unbalanced matches, but it can effectively differentiate the winning teams and losing in balanced matches. Our findings were in line with this study that identified free-throw percentage had no significant effect on the match result for MLR analysis, while this indicator was positively associated with the lower distribution (25th quantile, close matches) of final-match outcome. This may indicate that use of linear statistical approaches may underestimate the influence of free-throw percentage on the match outcome. However, differences between sexes may exist as a previous study for men’s basketball reported that free-throw percentage was not the KPI that can significantly affect the match result based on both linear and non-linear approaches (Zhang et al., 2020), In this regard, differences in motor abilities between women and men basketball players may be a plausible reasons for the different trends regarding the relationship between free-throw percentage and match outcome.

Offensive rebounds and assists showed significant positive effects on the match outcome for MLR analysis, but the results of QR analysis showed an opposite trend that offensive rebounds and assists had a significant impact on the upper and lower distribution of match result, respectively. This result may indicate that QR analysis as a non-linear statistical approach can provide more detailed information for the explanation of the relationships between technical performance indicators and match performance. Besides, the importance of offensive rebounds is well documented (Ibáñez et al., 2009b; García et al., 2014; Zhang et al., 2020) and has been verified again in this study, and assist as match action that directly impacts scoring is naturally closely related to the match outcome. Defensive rebounds and blocks, especially the defensive rebound, have been confirmed as the keys for teams’ success in previous studies (Gómez-Ruano et al., 2006; Summers, 2013; Leicht et al., 2017a). However, the results of MLR analysis demonstrated that defensive rebounds and blocks had no significant impact on match outcome, but the significant negative (defensive rebounds) and positive (blocks) effects on the match result were found using QR analysis. These disparities among two approaches may indicate that the effects of defensive rebounds and blocks on match outcome are sensitive. Therefore, caution should be paid by coaches on these indicators when developing the defensive strategies for women’s basketball competition.

Steals and fouls are widely used as performance indicators for the evaluation of defensive performance during match play. The execution of a successful steal can help the teams to recover of ball possession and more steals may contribute the probability of winning (Gómez-Ruano et al., 2013). Committing fouls will provide an easy scoring opportunity (i.e., free throws) for opponents and it has a negative impact on the match outcome. However, the current study identified that steals and fouls were not significantly associated with the match outcome, which is insistent with previous studies regarding both men’s (Leicht et al., 2017b) and women’s (Gómez-Ruano et al., 2006; Leicht et al., 2017a) basketball matches. This disparity may be due to differences in the application of statistical methods. Turnovers were the only indicator that showed significant relationships with match outcome for both MLR analysis and the entire range of quantiles of QR analysis which was in line with the result reported by Teramoto and Cross (2010) who found that turnover is a key predictor of teams’ success in the regular season games in the National Basketball Association (NBA). Others have also reported the importance of turnovers for basketball match success for both men’s and women’s matches in elite competitions (Olympic Games and FIBA Basketball World Cup) (Leicht et al., 2017a; Zhang et al., 2020). Passing errors were considered as the most common turnover in women’s basketball, and most of the turnovers happened during set plays (Fylaktakidou et al., 2011). The occurrence of a turnover is the result of good defensive decisions of opponents, leading to the loss of ball possession. Therefore, improving the ability to manage ball possession, and incorporating specific decision-making tasks into the training sessions with the consideration of specific situations (i.e., involving group-tactical situations) may potentially decrease the number of turnovers during the match play and increase the likelihood of team success.

## Conclusion

The current study has identified the key technical performance indicators that associated with match outcome in women’s basketball using linear and non-linear statistical methods. Our results indicated that QR analysis is more powerful when identifying the keys for teams’ success. The traditional linear modeling only describes the relationship between independent variables and the mean conditional distribution of dependent variables, while the QR analysis provides more detailed and practical information for understanding the relationships between technical performance indicators and various levels of distribution of match outcome. This may avoid the underestimation or overestimation of the effects of technical indicators on the match outcome. Additionally, our findings highlighted the KPIs in elite women’s basketball matches. The importance of two-point percentage, offensive rebounds, assists and turnover were confirmed by both MLR and QR. The significant effects of free-throw percentage, defensive rebounds and blocks on the match outcome were detected by MLR, while these were not the case in the results of QR. Three-throw percentage, steals and fouls were considered as non-critical indicators in women’s matches. This finding may allow coaches to get a better understanding of match success of women’s basketball matches and to control for technical–tactical strategies during match-play.

## Study Limitations

The limitations of the current study should be noted. First, the situational factors, such as match location and quality of opponent have not been considered in the analysis. Future research is recommended to take these situational variables and their interactions into account to improve the practical applications of the findings. Second, only one season was included in the analysis, the limited sample size could be one potential reason of the existing differences between this research and previous studies. Future research could expand the sample to identify the KPIs based on a longitudinal design across multiple seasons.

## Data Availability Statement

The raw data supporting the conclusions of this article will be made available by the authors, without undue reservation.

## Author Contributions

QY and WF: conceptualization. SZ: methodology, formal analysis, software, and visualization. QY: data collection and writing – original draft preparation. SZ, WF, and M-ÁG-R: writing – review and editing. M-ÁG-R: supervision. QY and WF: funding acquisition. All authors have read and agreed to the published version of the manuscript.

## Conflict of Interest

The authors declare that the research was conducted in the absence of any commercial or financial relationships that could be construed as a potential conflict of interest.

## Footnotes

1 https://www.cba.net.cn
